# SG-APSIC1023: Contamination of the geriatric medicine outpatient rehabilitation gym environment and the effectiveness of our current disinfection methods with patient hand hygiene practices

**DOI:** 10.1017/ash.2023.40

**Published:** 2023-03-16

**Authors:** Sabrina Toh, Foo Meow Ling, Li Dun Li, Calvin Loh, Toylyn Lee, Angeline Seah

**Affiliations:** Khoo Teck Puat Hospital, Singapore; Singapore, Khoo Teck Puat Hospital, Singapore; Singapore, Khoo Teck Puat Hospital, Singapore; Khoo Teck Puat Hospital, Singapore; Khoo Teck Puat Hospital, Singapore; Khoo Teck Puat Hospital, Singapore

## Abstract

**Objectives:** To quantify the microorganism burden of rehabilitation gym equipment surfaces as well as to assess the effectiveness of patient’s practice of hand hygiene and our current disinfection methods to reduce burden and transmission of microorganisms during rehabilitation sessions. **Methods:** A prospective study of environmental contamination using microbiology culture in Khoo Teck Puat Hospital Geriatric Medicine Outpatient Rehabilitation Gym. **Results:** For both the control and intervention group, the total aerobic bacterial count on the gym equipment after patient use is significant and increase up to 360 CFU per swab. In the control and intervention groups, the total aerobic bacterial counts on the gym equipment before patients’ use were negligible (<10 CFU per swab). The total aerobic bacterial count of the equipment remained negligible (<10 CFU per swab) after patient use and immediate disinfection. We detected discrepancies between the results of the total aerobic bacterial count after patient use between the control and intervention groups. **Conclusions:** Outpatient rehabilitation gyms are potential reservoirs of microorganisms, which may further contribute to the transmission of healthcare-associated pathogens. In this study, an intervention in which cleaning equipment was wiped with alcohol wipes was effective in reducing microorganism transmission in the rehabilitation gym environment and should be considered as part of our infection control strategy. The additional step of involving our patients in using hand rub before the start of their therapy sessions can provide additional benefit in reducing microorganism transmission only if patients adhere to the World Health Organization (WHO) recommended 7 steps of proper hand rub. Good patient education on hand hygiene is equally as important as that for healthcare professionals to control environmental contamination.

